# C/EBP Homologous Protein (CHOP) Activates Macrophages and Promotes Liver Fibrosis in *Schistosoma japonicum*-Infected Mice

**DOI:** 10.1155/2019/5148575

**Published:** 2019-12-01

**Authors:** Mengyun Duan, Yuan Yang, Shuang Peng, Xiaoqin Liu, Jixin Zhong, Yurong Guo, Min Lu, Hao Nie, Boxu Ren, Xiangzhi Zhang, Lian Liu

**Affiliations:** ^1^Department of Medical Imaging, Medical School of Yangtze University, Jingzhou 434023, China; ^2^Department of Radiology, Renmin Hospital of Wuhan University, Wuhan 430060, China; ^3^Cardiovascular Research Institute, Case Western Reserve University, Cleveland, OH 44106, USA; ^4^Department of Pathogenic Biology, Medical School of Yangtze University, Jingzhou 434023, China; ^5^Clinical Molecular Immunology Center, Medical School of Yangtze University, Jingzhou 434023, China; ^6^Department of Pharmacology, Medical School of Yangtze University, Jingzhou 434023, China

## Abstract

CCAAT/enhancer-binding homologous protein (CHOP), a transcriptional regulator induced by endoplasmic reticulum stress (ER stress) is a pivotal factor in the ER stress-mediated apoptosis pathway. Previous studies have shown that CHOP is involved in the formation of fibrosis in a variety of tissues and is associated with alternative macrophage activation. The role of CHOP in the pathologic effects of liver fibrosis in schistosomiasis has not been reported, and underlying mechanisms remain unclear. This study is aimed at understanding the effect of CHOP on liver fibrosis induced by *Schistosoma japonicum* (*S. japonicum*) in vivo and clarifying its mechanism. C57BL/6 mice were infected with cercariae of *S. japonicum* through the abdominal skin. The liver fibrosis was examined. The level of IL-13 was observed. The expressions of CHOP, Krüppel-like factor 4 (KLF4), signal transducer and activator of transcription 6 (STAT6), phosphorylation STAT6, interleukin-13 receptor alpha 1 (IL-13R*α*1), and interleukin-4 receptor alpha (IL-4R*α*) were analysed. The eosinophilic granuloma and collagen deposition were found around the eggs in mice infected for 6 and 10 weeks. IL-13 in plasma and IL-13R*α*1 and IL-4R*α* in liver tissue were significantly increased. The phosphorylated STAT6 was enhanced while Krüppel-like factor 4 (KLF4) was decreased in liver tissue. The expression of CHOP and colocalization of CHOP and CD206 were increased. Overall, these results suggest that CHOP plays a critical role in hepatic fibrosis induced by *S. japonicum*, likely through promoting alternative activation of macrophages.

## 1. Introduction

Schistosomiasis, a common parasitic disease caused by parasitic flatworms called schistosomes, is mainly prevalent in developing countries and is severely impacting people's health, causing enormous health and socioeconomic burdens to mankind. 200 million people worldwide are still under threat from schistosomiasis in 2016 [1]. Epidemiological surveys show that schistosomiasis endemic areas are distributed in 178 counties in 11 provinces in China's mountain areas [2]. China is one of the primary epidemic areas of *S. japonicum*. There are still 37,601 cases of schistosomiasis in China, and 259 million people are threatened by schistosomiasis in 2016 [3].

Hepatic fibrosis is a significant hallmark during the progression from *S. japonicum* infection-induced schistosomiasis to the end-stage liver disease [4–6]. Macrophages play an indispensable role in the fibrosis process of different tissues or organs, which not only participates in early inflammatory reactions but also secretes various inflammatory factors to participate in the body's immune response [7–12]. At present, the mainstream view is that macrophages can be divided into classically activated macrophages mainly stimulated by LPS or IFN-*γ* and an alternatively activated phenotype mainly stimulated by IL-4 or IL-13 [13]. These classically activated macrophages are also known as M1 macrophages, and the alternatively activated macrophages are also known as M2 macrophages [10]. A large number of M2 macrophages infiltrate in the liver during the course of *S. japonicum* infection, and animal studies have shown that M2 macrophages may exhibit as a novel target for the prevention and treatment of fibrosis [14]. M2 macrophages produce various factors during the development of fibrosis such as arg-1 and FIZZ1, which aggravate Th2 immunity [15]. Furthermore, arg-1 can hydrolyze l-arginine into proline and polyamine, promoting the synthesis of collagen and the occurrence of fibrosis [16].

CHOP is also known as CHOP 10, DDIT 3, or GADD153; its promotion in the process of fibrosis is increasingly confirmed, despite the well-recognized role of CHOP in facilitating apoptosis, unfolded protein response (UPR), and integrated stress response (ISR). Moreover, CHOP deficiency can alleviate pulmonary fibrosis [17], renal fibrosis [18], and liver fibrosis [19], accompanied by a decreased polarization of M2 macrophages. However, it is not clear if CHOP is involved in liver fibrosis during schistosomiasis. We noted that CHOP and the M2 macrophage marker CD206 were spatially colocalized in the liver tissue of mice infected with schistosomiasis. Therefore, we hypothesized that CHOP mediates the production of M2 macrophages to promote pathological changes associated with fibrotic development. To validate this hypothesis, we established a C57BL/6 mouse as an experimental schistosomiasis model for *S. japonicum* to analyse how CHOP is associated with liver fibrosis formation and possible underlying mechanisms.

## 2. Materials and Methods

### 2.1. Animal and *S. japonicum* Infections

Eight-week-old C57BL/6 mice, weighting 22 ± 2 g, were purchased from the Hubei Provincial Center for Disease Control and Prevention (Wuhan, Hubei Province, China), and cercariae of *S. japonicum* were bought from Jiangsu Provincial Center for Disease Control and Prevention (Nanjing, Jiangsu Province, China). 100 mice were randomly divided into the control group and the infection group, of which 30 were in the control group and 70 in the infection group. Mice were infected with cercariae of *Schistosoma japonicum* to establish a hepatic fibrosis model. When the room temperature is 26°C, the *Oncomelania hupensis* were placed in purified water, and then a microscope (DM 1000, Leica, Germany) was used to count the number of cercariae. We used the abdominal patch method to infect mice. The abdomen hairless skin was exposed to the cercariae for 15-20 minutes under natural light (50 ± 2). The mice were euthanized 6 or 10 weeks after infection, and liver specimens were collected.

### 2.2. Liver Index

Mice were euthanized 6 and 10 weeks after undergoing infection of cercariae of *S. japonicum* and the weight of wet liver (*A*) and body weight (*B*) were recorded. The acquired data were expressed as the wet liver weight divided by the total body weight times 100. Liver/body weight ratio  = *A*/*B* × 100%.

### 2.3. Histology

Liver tissues were fixed in 4% formalin and embedded in paraffin. Then, these histological sections were cut at 5 *μ*m. Hematoxylin-eosin (HE) staining was used to analyse the area of granuloma. Masson staining was used to analyse the degree of collagen deposition. Images of six random microscopic fields of blue-stained collagen fibres in the liver section of each mouse were recorded using an inverted microscope (Olympus, Olympus DP27, Japan) and then digitized and analysed on Image-Pro Plus software 6.0 as previously described. A Masson Stain Kit was offered by Wuhan Servicebio Technology Company (BA-4079A, BASO, Zhuhai, China).

### 2.4. Immunohistochemistry

For immunostaining, the slices were placed in an EDTA pH 9.0 buffer and microwaved for antigen repair, with medium heat for 8 minutes, and low heat for 8 minutes before power failure. After natural cooling, wash the slices with PBS 3 times for 5 minutes each. Nonspecific proteins were blocked with 3% BSA for 30 minutes. The sections were then probed with a mouse-derived anti-CHOP antibody (Santa Cruz, United States; 1 : 50) and a rabbit-derived anti-fibronectin antibody (Abcam, the United Kingdom; 1 : 500) at 4°C overnight, followed by incubation with an HRP-labeled anti-mouse-IgG and an HRP-labeled anti-rabbit-IgG secondary antibody (SeraCare, United States; 1 : 100) at room temperature for 50 minutes.

### 2.5. Immunofluorescence

Procedures for liver tissue fixation, dehydration, and embedding were described above. For immunofluorescence, the slices were placed in an EDTA pH 8.0 buffer for microwave repair, and power was cut off after 8 minutes of low heat. The washing and nonspecific protein blocking steps are as described above. The sections were then probed with a rabbit-derived anti-CHOP antibody (Bioss, Beijing, China; 1 : 200) and a rabbit-derived anti-CD206 antibody (Proteintech, Wuhan, China; 1 : 200) at 4°C overnight, followed by incubation with an FITC-labeled anti-rabbit-IgG and an CY3-labeled anti-rabbit-IgG secondary antibody (Hundred Thousand Biological Technology, Wuhan, China; 1 : 100) at room temperature for 50 minutes. Sections incubated with normal IgG were used as a negative control. Immunofluorescence images were acquired using a scanning sequential mode to avoid bleed-through effects with a fluorescence microscope (Eclipse CI, Nikon, Japan), and images were processed using the MicroPublisher (Q-IMAGING, Canada).

### 2.6. RNA Isolation and RT-qPCR

Total RNA was extracted from mice liver tissue homogenate with TRIzol (Invitrogen, USA) and quantified by NanoPhotometer® NP80 (Implen, Germany). The total RNA (1 *μ*g) was reverse transcribed to cDNA with a PrimeSecript™ RT reagent kit (Takara RR047A, Japan) and incubated at 42°C for 2 min to remove genomic DNA then incubated at 37°C for 15 min and 85°C for 5 s. Real-time quantitative PCR (RT-qPCR) was carried out using a three-step approach at 95°C for 30 s, followed by 95°C for 5 s, 60°C for 30 s, and 72°C for 30 s according to the specification for the SYBR® Premix Ex Taq™ (Tli RNaseH Plus, Takara, Japan) on an Applied Biosystems 7500 Real-Time-PCR System (Life Technologies, USA). All the primers were synthesized by Sangon Biotech (Wuhan, China), and the primer sequence is listed in Table 1. Gene expression was normalized to GAPDH using the *^ΔΔ^*Ct method.

### 2.7. Western Blot

Liver tissues were lysed in a RIPA lysis buffer (P0013b, Beyotime Biotechnology, China), and proteins were quantified by a BCA protein assay kit (Beyotime Biotechnology, China). Equivalent amounts (40 *μ*g) of total protein were separated by 12% SDS-PAGE and transferred to a PVDF membrane (Immobilon-P, Germany). The membrane was subsequently blocked with 5% skim milk in TBST solution for 2 h at room temperature and incubated at 4°C overnight, using the following primary antibodies: KLF4 (1 : 500; Abcam, Cambridge, MA, USA), GAPDH (1 : 1000; CST, USA), and CHOP (1 : 500; Santa Cruz, United States). HRP-conjugated secondary antibodies (1 : 10000) were next applied for 1 h at room temperature. The antigen-antibody complexes were detected by a Pierce ECL substrate kit (MULTI SCIENCES, China). Specific bands were scanned and quantified by Quantity One software (Bio-Rad, USA). GAPDH was used as the loading control.

### 2.8. ELISA

The IL-13 levels in the serum of the mice were determined by ELISA. The sera were assayed for IL-13 with mouse IL-13 ELISA kits (Elabscience, Wuhan, China). The procedures were executed according to the instruction manuals for the kits. Sterile PBS was used as a control.

### 2.9. Statistical Analysis

All data are presented as mean ± standard error of mean (SEM). Two-way ANOVA was used for comparison of different treatment time groups, followed by Tukey's multiple comparison test or Sidak's multiple comparison test. All statistical tests were performed using GraphPad Prism 6.0 (GraphPad Software, Inc.). Data was considered statistically significant only when *P* < 0.05.

## 3. Results

### 3.1. *S. japonicum* Infection Caused Liver Fibrosis

**Figure 1:**
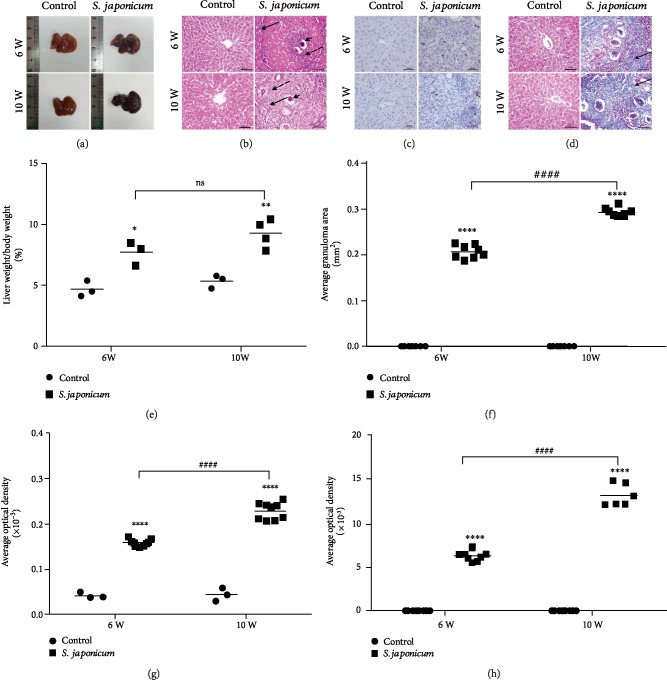
Liver fibrosis is aggravated by *S. japonicum* infection. The animals were infected with 50 ± 2*S. japonicum* cercariae. HE staining, Masson trichrome staining, and immunohistochemistry were used to observe the pathological changes and collagen deposition of liver tissue. (a) The morphology changes on the liver of *S. japonicum* infection in mice. (b) Liver tissues were stained with HE. Arrows indicate granulomatous lesions and arrowheads indicate schistosome eggs (magnified ×200). (c) The protein expression of fibronectin detected by immunohistochemistry (magnified ×400). (d) Liver tissues were stained with Masson's trichrome staining (magnified ×200). (e) Liver/body ratio of the infection group and the control group after *S. japonicum* cercariae. (f) Areas of single egg granulomas. (g) The quantitative analysis of fibronectin in liver tissues. (h) Average optical density of the collagen deposition in liver tissues. Data were presented as mean ± SEM of 6-8 mice. ^∗^*P* < 0.05 and ^∗∗^*P* < 0.01 vs. the control. ^#^*P* < 0.05 and ^##^*P* < 0.01 vs. the 6-week infection group.

Compared with the control group, the liver/body weight ratio (LBWR) of mice infected with *S. japonicum* was significantly increased (Table 2, Figure 1(e)). Liver weights, etc. were normalized by body weight. The normal mice liver was displayed as ruddy with a smooth surface and bright red, whereas the liver of the infected mice appeared as dark red and had many small white granulomatous nodules on the surface (Figure 1(a)).

To study the liver pathology of mice infected with *S. japonicum*, the fixed liver tissues were stained with HE or Masson's trichrome (Figures 1(b) and 1(d)). The deposition of *S. japonicum* eggs and granulomas in the hepatic tissue from *S. japonicum*-infected mice was significantly elevated compared with that from the control mice (Figures 1(b) and 1(f)). The results showed that the lobule structure was intact; radiated liver cell cords were visible in the normal group (Figure 1(b)). After 6 weeks of infection, a large number of granular immune cells (see black dotted arrows) infiltrated the area around the eggs (see the short black arrow), surrounded by fibrous vascular tissue bands (see black solid arrow). More eggs (see the short black arrow) were deposited in the liver at 10 weeks, surrounded by a large amount of granulomatous tissue (see black dotted arrows) and entangled by fibrous tissue (see black solid arrow). Compared with the control group, the average granuloma area increased significantly after 6 and 10 weeks after *S. japonicum* infection (Figure 1(f)).

Fibrotic collagen deposition, dyed blue, around the *S. japonicum* egg granulomas was evaluated by Masson's trichrome (Figure 1(d)). It showed no blue staining in the liver tissue of the control group, and a few fibres began to appear around the eggs after 6 weeks of infection (see black solid arrow). In addition, 10 weeks after infection, a large amount of fibres were deposited in the liver tissue (see black solid arrow), surrounding the egg deposition site (Figure 1(d)). We further analysed the average optical density (AOD) to reflect the expression level of collagen fibres. Compared with the control group, the expression of collagen fibres increased significantly after 6 and 10 weeks of *S. japonicum* infection (Figure 1(h)). Besides, fibronectin, a marker of liver fibrosis, increased significantly after infection with *S. japonicum* compared to the uninfected group, and fibronectin deposition was aggravated with prolonged infection time (Figures 1(c) and 1(g)).

### 3.2. The Expression of CHOP in the Liver of Mice Increased in the Liver of *S. japonicum*-Infected Mice

**Figure 2:**
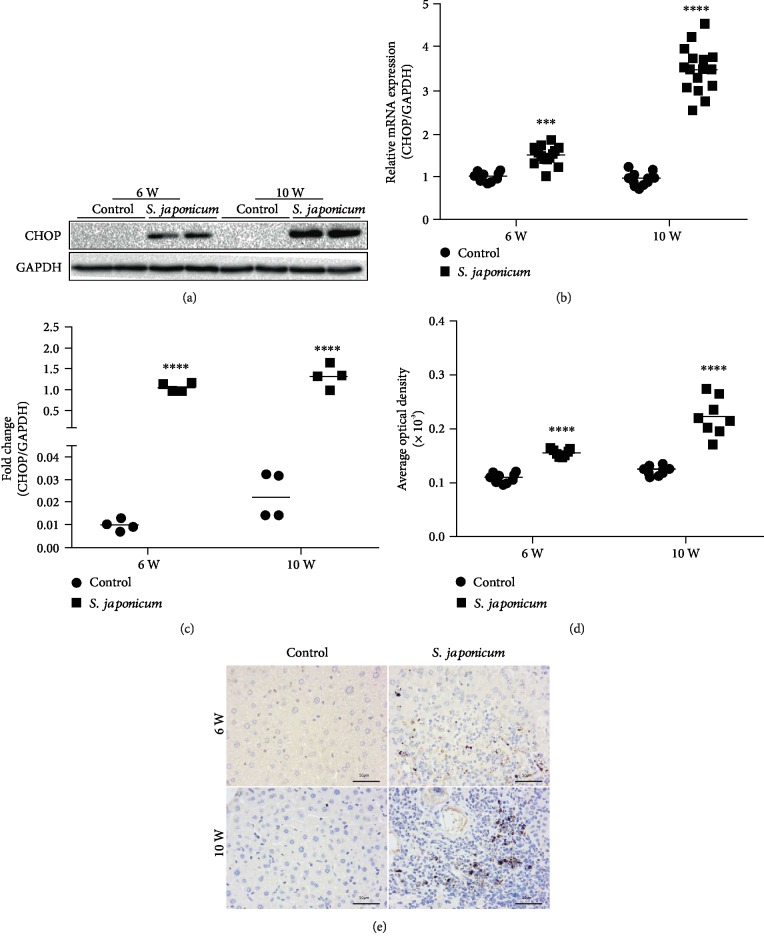
Expression of CHOP in transcription and protein levels increased after *S. japonicum* cercariae infection in murine liver homogenates. Mice were treated with *S. japonicum* cercariae or sterile 9% physiological saline; after being administrated for 6 and 10 weeks, 6-8 mice were euthanized. Real-time PCR, Western blot, and immunohistochemical assays were performed to detect the expression of CHOP in the liver homogenate. (a) The protein expression of CHOP detected by WB. (b) The mRNA expression quantitative analysis of CHOP in the liver homogenate. (c) The protein expression quantitative analysis of CHOP in the liver homogenate. (d) The quantitative analysis of CHOP in liver tissues. (e) The protein expression of CHOP detected by immunohistochemistry (magnified ×400). Data were presented as mean ± SEM of 6-8 mice. ^∗^*P* < 0.05 and ^∗∗^*P* < 0.01 vs. the control. ^#^*P* < 0.05 and ^##^*P* < 0.01 vs. the 6-week infection group.

Compared with the control group, the mRNA expression of CHOP increased by 1.5- and 3.5-folds after 6 and 10 weeks of infection with *S. japonicum*, respectively (Figure 2(b)). To further confirm subcellular localization of CHOP and protein level expression, immunohistochemistry and WB were performed to detect the CHOP expression. Consistent with the RT-qPCR results, WB and immunohistochemistry results showed that the CHOP expression increased after infection with *S. japonicum*, compared with the age-matched uninfected group, and the expression after 10 weeks of infection was higher than that after 6 weeks of infection (Figures 2(a) and 2(c)–2(e)).

### 3.3. The STAT6 Signal Pathway Is Activated and the KLF4 Expression Is Reduced following *S. japonicum* Infection

**Figure 3:**
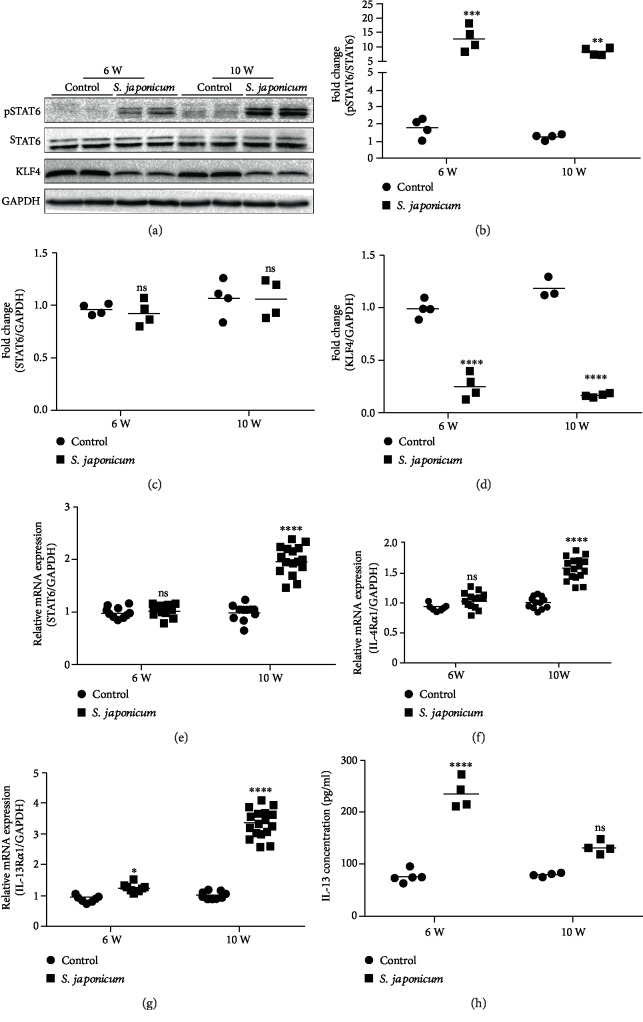
*S. japonicum* significantly increased the protein level of pSTAT6 and reduced the protein level of KLF4. (a) The protein expression of pSTAT6, STAT6, and KLF4 detected by Western blots. (b) Relative protein levels of pSTAT6/STAT6 were analysed. (c, d) Relative protein levels of STAT6 and KLF4 in the liver homogenate was analysed using GAPDH as a loading control. (e–g) Relative mRNA levels of STAT6, IL-4R*α*, and IL-13R*α*1 in the liver homogenate. (h) The IL-13 levels in the serum. Data are presented as mean ± SEM of 6-8 mice. ^∗^*P* < 0.05 and ^∗∗^*P* < 0.01 vs. the control. ^#^*P* < 0.05 and ^##^*P* < 0.01 vs. the 6-week infection group.

The phosphorylation of STAT6 approximately increased by 7- and 6-folds after 6 and 10 weeks of infection with *S. japonicum*, respectively, compared to the age-matched uninfected group (Figures 3(a) and 3(b)). At the same time, the expression of the KLF4 protein in the liver homogenate was also detected. The results showed that the expression of KLF4 approximately reduced by 4- and 7-folds after 6 and 10 weeks of infection with *S. japonicum*, respectively, compared to the age-matched uninfected group (Figures 3(a) and 3(d)). The mRNA expression of STAT6, IL-4R*α*, and IL-13R*α*1 increased approximately 1.5-3-folds after 10 weeks of infection with *S. japonicum*, and there were no obvious differences between the uninfected group and the infected group after 6 weeks of infection with *S. japonicum* except IL-13R*α*1 (Figures 3(a) and 3(e)–3(g)). Coincidently, the results showed that the expression of IL-13 in the serum in the infected group was significantly increased compared with the uninfected group (Figure 3(b)). In general, our data indicate that *S. japonicum* infection increases pSTAT6, IL-4R*α*, IL-13R*α*1, and IL-13 expression and reduces KLF4.

### 3.4. The Expression of CHOP Increased Significantly in M2 Macrophages after *S. japonicum* Infection

**Figure 4:**
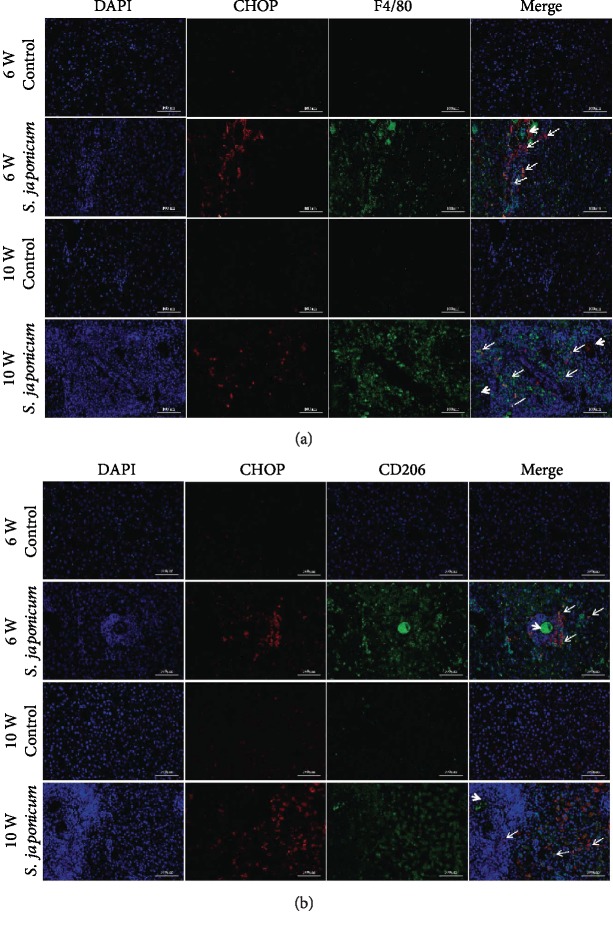
*S. japonicum* significantly increased the expression of CHOP in M2 macrophages. Immunofluorescence assays were performed to detect the colocalization of CD206 and CHOP. (a) Coimmunostaining of CHOP and F4/80 in liver tissues (magnified ×200). (b) Coimmunostaining of CHOP and CD206 in liver tissues (magnified ×200). Data were presented as mean ± SEM of 6-8 mice. ^∗^*P* < 0.05 and ^∗∗^*P* < 0.01 vs. the control. ^#^*P* < 0.05 and ^##^*P* < 0.01 vs. the 6-week infection group.

To confirm the expression of CHOP in specific cell types, we used immunofluorescence to detect the cellular localization of CHOP in liver tissues. The results showed that CHOP was expressed in F4/80^+^ macrophages. One or several eggs (see white dotted arrows) are surrounded by CHOP^+^ and F4/80^+^ macrophages (see white solid arrow), and the number of CHOP^+^ and F4/80^+^ coimmunostaining cells was significantly increased in a time-dependent manner (Figure 4(a)). CHOP and CD206 immunofluorescence assays showed that these macrophages were characterized by the M2 subtype. Eggs (see white dotted arrows) are surrounded by CHOP^+^ and CD206^+^ macrophages (see white solid arrow) (Figure 4(b)). The infiltration number of M2 macrophages in mice 10 weeks after infection was larger than that in mice 6 weeks after infection. In general, our results suggest that CHOP may be involved in the pathological process of fibrosis by regulating the activation of macrophages.

## 4. Discussion

*S. japonicum* infection often causes granulomatous inflammation at the site of egg deposition, leads to dysregulation of the immune system, activates hepatic stellate cells, and even causes persistent liver fibrosis [20]. The main pathological feature of liver fibrosis is the excessive deposition of ECM in tissues, which leads to excessive protein synthesis and disturbance of endoplasmic reticulum homeostasis [21–23]. CHOP is considered to be one of specific and convergent transcription factors of ER stress, and its activation is generally regulated at the transcriptional level [24]. Yu et al. [25] showed that taurine attenuating the hepatic pathologic features in mice infected with *S. japonicum* was related to the regulation of ERS. Recent studies have focused on the functions of CHOP on the formation of organ fibrosis in different disease models [17, 18, 26–31]. CHOP expression was significantly increased in many liver fibrosis models induced by different factors. These factors include drugs (such as carbon tetrachloride [32] and thioacetamide [33]), metabolic abnormalities (such as BDL [30, 34] and MCD diet [28, 35]), and alcohol [36–38]. Liver fibrosis is reduced and CHOP expression is reduced after treatment [19, 39]. Tauroursodeoxycholic acid (TUDCA), a hydrophilic bile acid, is a drug that inhibits the expression of CHOP, which alleviates liver fibrosis caused by cholestasis [22] and inhibits pulmonary fibrosis induced by bleomycin (BLM) [40]. In addition, the use of CHOP-deficient mice suggests that CHOP deficiency can reduce liver fibrosis caused by bile duct ligation [30, 34], cholestasis [30], or MCD diet [28] and retard renal fibrosis caused by diabetic nephropathy [21, 41]. Furthermore, in the human liver, the expression of CHOP is enhanced as the progression of nonalcoholic steatohepatitis to HCC [28]. However, the role of CHOP in liver fibrosis of *S. japonicum* is still not well understood. In the present study, the level of CHOP expression in infected mice was augmented compared to uninfected mice, suggesting that CHOP plays a critical role in hepatic fibrosis induced by *S. japonicum* [35].

Researchers found that glycoproteins such as IPSE (SmEP-25) [42], omega-1 [43], and kappa-5 [44] secreted by *S. mansoni* eggs and Sm29 [45] secreted by S. *haematobium*stimulate Th2 cytokine production in mice. For *S. japonicum*, egg secretory proteins (ESP) instead of IPSE, omega-1, and kappa-5 played a central role in driving the development of the immune response to the Th2 pattern because it was predicted to stimulate the production of IL-1, IL-3, IL-5, IL-4, and IL-13 [46]. Th2 response is supported by alternatively activated antigen presenting cells (APCs). A growing number of researches payed attention to the role of M2 macrophages in the pathological process of schistosomiasis, especially liver fibrosis [47–50]. One or several eggs are surrounded by immune cells (mainly including alternately activated macrophages, Th2 cells, and eosinophils) and the extracellular matrix (ECM), protecting the host tissue from the toxins [51]. Gong and his team found that excretory secretion of antigen (ES) from *Schistosoma japonicum* eggs can activate macrophages to exhibit enhanced M2b polarization [47]. It has been reported that reducing or even eliminating mice macrophages can reduce organ fibrosis [52, 53], while M2 macrophage adoptive transplantation can aggravate organ fibrosis in mice [54, 55]. Moreover, the decrease in the infiltration of M2 macrophages can be observed in the CHOP^−/−^ animal fibrosis model [18, 56, 57]. In our study, IL-13 in the serum was significantly increased in the infected group and immunofluorescence of the liver tissue showed a significant increase in the number of CHOP^+^ CD206^+^ macrophages in the infected group, compared with the uninfected group. Based on the above findings and previous work in our laboratory, we speculate that CHOP may mediate the polarization of M2 macrophages and participate in the formation of hepatic granuloma and fibrosis caused by *S. japonicum*. However, what molecular mechanisms are involved in M2 macrophage polarization?

STAT6 is an important regulatory transcription factor for M2 macrophage polarization [58]. Increasing evidence suggests that STAT6-mediated M2 macrophage polarization activation contributes to tissue fibrosis [59–62]. Corilagin reduces liver fibrosis induced by *S. japonicum* infection via reducing the expression of IL-13/STAT6 signal pathway-related molecules in alternative activated macrophages [48]. In our study, pSTAT6, IL-4R*α*, and IL-13R*α*1 were significantly increased following infection with *S. japonicum*. Research by Adedokun and his colleagues suggests that the IL-4 promoter region is a predisposing factor for schistosomiasis and is critical in regulating the disease burden and that carriers of the rs2243250 T/T mutation are more severely affected. With the deletion of STAT6, the rs3024974T/T variant among infected children indicated the need for the STAT6 promoter gene in provoking schistosomiasis susceptibility in Nigeria [63]. With the application of mutant mice, the role of STAT6 activation in the study of fibrosis mechanisms is demonstrated. Moreover, a number of studies confirmed that STAT6 plays a role in different organs. STAT6 deficiency inhibits accumulation of bone marrow-derived fibroblasts, formation of myofibroblasts, expression of ECM proteins, and deposition of collagen in obstructed kidneys [64]. Compared with wild-type (WT) mice, the lungs from STAT6^−/−^ mice show inhibition of acute inflammation and reduction of fibrotic diseases after administration of multiwalled carbon nanotubes (MWCNT) [65]. The study found that IL-4 interacts with type I and type II IL-4Rs to induce downstream signalling pathways, including the JAK3-STAT6 signalling pathway [66]. It indicates that the IL-4/IL-4R*α* pathway is closely related to fibrotic diseases. Consistent with this, IL-4R*α* deficiency inhibits STAT6 activation, monocyte to fibroblast transformation, and ECM protein production following IL-4 treatment [67]. In addition, IL-4R*α* has profibrotic effects through activated macrophages during mouse skin repair [68]. Furthermore, CHOP deletion significantly reduced IL-4-induced STAT6 phosphorylation and IL-4R*α* expression [69]. These data suggest that CHOP may regulate M2 macrophage polarization and mediated fibrosis via the IL-4/STAT6 signalling pathway.

It is reported that KLF4 is essential for IL-4-mediated macrophage M2 polarization and synergizes with STAT6 to promote M2 macrophage polarization [70]. We considered whether KLF4 is involved in the model of *S. japonicum* liver fibrosis. However, our results found that the KLF4 protein was reduced in liver homogenates in this model. Consistent with this, in the liver fibrosis model, KLF4 expression was decreased in hepatic stellate cells [71]. In addition, according to Men and his colleague's research, KLF4 was reduced in activated hepatic stellate cells (HSCs) and cirrhotic liver tissues and KLF4 deficiency promote HSC activation [72]. Another study suggests that other mechanisms independent of IL-4 may also regulate the M2 phenotype [73]. Therefore, whether KLF4 is involved in macrophage polarization in the *S. japonicum* model is an important issue that needs further study. At the same time, studies have found that KLF4 expression is decreased in patients with liver fibrosis and rat liver, and the results show that decreased expression of KLF4 can activate HSCs [74]. HSC activation is an important process of liver fibrosis, which can secrete a large amount of ECM and high expression of *α*-SMA and collagen to promote the development of fibrosis [75]. KLF4 overexpression inhibits the expression of *α*-SMA and collagen while inhibiting the proliferation of HSCs [71, 74]. In the early stage of liver fibrosis, the expression of KLF4 is higher to maintain the stability of activated HSCs [76] and inhibit the differentiation and activation of HSCs [71]. With the progression of liver fibrosis, the expression of KLF4 is gradually inhibited. It has also been reported that CHOP inhibits the expression of KLF4 at the transcriptional and translational levels by downregulating ATF4. When the CHOP gene is deleted, the expression of KLF4 is involved in the inhibition of cell proliferation [77]. In C57BL/6 mice, iron overload induced decreased KLF4 expression and liver fibrosis [78]. In summary, we speculate that KLF4 may play different roles in different disease models or organs, and KLF4 expression in different cell types may have different effects on the formation of liver fibrosis. Our study found that KLF4 reduction is involved in the process of liver fibrosis induced by *S. japonicum*. Combined with our observations, CHOP may promote liver fibrosis caused by *S. japonicum* by reducing the expression of KLF4 and activating M2 macrophages.

In conclusion, our results indicate that CHOP plays an important role in the progression of liver fibrosis caused by *S. japonicum*. We speculate that CHOP may be a key gene in the pathogenesis of *S. japonicum*-induced liver fibrosis because it has a wide range of effects in vivo. Further understanding of CHOP-mediated liver fibrosis and its regulation with hepatic macrophages may provide new ideas for the treatment of liver fibrosis in schistosomiasis. Next, we will further explore the regulatory mechanisms through gene knockout animals and cell experiments. Therefore, our future research focuses on testing the potential of CHOP antagonists to improve liver fibrosis induced by *S. japonicum*. In this case, the damaging effects of CHOP deficiency in the liver should be warily treated.

## Figures and Tables

**Table 1:** Sequences of real-time PCR primers used throughout.

Primer	Direction	Sequence	Temperature	Length
CHOP	Forward	5′-TATCTCATCCCCAGGAAACG-3′	60	219
	Reverse	5′-GGGCACTGACCACTCTGTTT-3′		
STAT6	Forward	5′-CTCTGTGGGGCCTAATTTCCA-3′	60	135
	Reverse	5′-CATCTGAACCGACCAGGAACT-3′		
IL-4 receptor *α*	Forward	5′-GCTGCTGACCTGGAATAACCT-3′	60	181
	Reverse	5′-CGCCGTATAGTAGACCCCTG-3′		
IL-13 receptor *α*1	Forward	5′-TCAGCCACCTGTGACGAATTT-3′	60	101
	Reverse	5′-TGAGAGTGCAATTTGGACTGG-3′		
GAPDH	Forward	5′-GGTTGTCTCCTGCGACTTCA-3′	60	183
	Reverse	5′-TGGTCCAGGGTTTCTTACTCC-3′		

**Table 2:** Effect of *S. japonicum* infection on the body weight, liver weight, and LBWR of mice.

Infection time (weeks)	Body weight (g)		Liver weight (g)		Liver/body weight ratio (g)	
	Control group	Model group	Control group	Model group	Control group	Model group
6	20.8 ± 1.1	25.3 ± 2.8	0.97 ± 0.11	1.92 ± 0.1	4.65 ± 0.61	7.68 ± 0.97
10	21.1 ± 0.9	26.1 ± 0.4	1.12 ± 0.07	2.32 ± 0.3	5.33 ± 0.48	9.27 ± 1.16

Changes of liver index (%) in control and infected mice at different time points after *S. japonicum* infection. Data were presented as mean ± SEM of 6-8 mice.

## Data Availability

Our data used to support the findings of this study are all included within the article

## Abbreviations

CHOP: C/EBP homologous protein
ER stress: Endoplasmic reticulum stress
UPR: Unfolded protein response
ISR: Integrated stress response
S. *japonicum*: *Schistosoma japonicum*
STAT6: Signal transducer and activator of transcription 6
pSTAT6: Phosphorylation signal transducer and activator of transcription 6
IL-4 R*α*: Interleukin-4 receptor alpha
IL-13R*α*1: Interleukin-13 receptor alpha 1
KLF4: Krüppel-like factor 4
LBWR: Liver/body weight ratio
APC: Antigen presenting cells
ECM: Extracellular matrix
AOD: Average optical density
ES: Excretory secretion
*γ*T3: Gamma-tocotrienol
UUO: Unilateral ureteral obstruction
BDL: Bile duct ligation
RT-qPCR: Real-time quantitative PCR
WB: Western blot
HE: Hematoxylin-eosin
EDTA: Ethylenediaminetetraacetic acid
BSA: Bull serum albumin
PBS: Phosphate buffer solution
TBST: Tris-buffered saline Tween
HRP: Horseradish peroxidase
WT: Wild type
MWCNT: Multiwalled carbon nanotubes
ESP: Egg secretory proteins
HSCs: Hepatic stellate cells.

## Supplementary Materials

Supplementary Materials Supplementary Figure 1: H&E staining and Masson staining of liver tissue of mice. (A) Liver tissues were stained with HE in uninfected group mice. (B) Liver tissues were stained with HE after 10 weeks of *S. japonicum* infection. (C) Liver tissues were stained with Masson's trichrome staining after 10 weeks of *S. japonicum* infection. The thin black arrow indicates fibres and the thick black arrow indicates schistosome eggs (magnified 50 times, 100 times, 200 times, and 400 times from left to right). Supplementary Figure 2: negative control for immunofluorescence. (A) Negative control of liver immunofluorescence in uninfected group mice. (B) Negative control of liver immunofluorescence after 10 weeks of *S. japonicum* infection (magnified by 200 times (left) and 400 times (right)). Supplementary Figure 3: negative control for immunohistochemistry. (A) Negative control of liver immunohistochemistry in uninfected group mice. (B) Negative control of liver immunohistochemistry after 10 weeks of *S. japonicum* infection (magnified by 200 times (left) and 400 times (right)).

## References

[1] (2017). Global, regional, and national incidence, prevalence, and years lived with disability for 328 diseases and injuries for 195 countries, 1990-2016: a systematic analysis for the Global Burden of Disease Study 2016. *The Lancet*.

[2] Liu Y., Zhou Y.-B., Li R.-Z., Zhou X. N., Li S. Z., Utzinger J., Bergquist R. (2016). Schistosomiasis in The People's Republic of China - From Control to Elimination. *Advances in Parasitology*.

[3] Li-Juan Z., Zhi-Min X., Si-Min D. (2018). Endemic status of schistosomiasis in People’s Republic of China in 2017. *Zhongguo xue xi chong bing fang zhi za zhi = Chinese journal of schistosomiasis control*.

[4] Kamdem S. D., Moyou-Somo R., Brombacher F., Nono J. K. (2018). Host regulators of liver fibrosis during human schistosomiasis. *Frontiers in Immunology*.

[5] Aufses A. H., Schaffner F., Rosenthal W. S., Herman B. E. (1959). Portal venous pressure in "pipestem" fibrosis of the liver due to schistosomiasis. *The American Journal of Medicine*.

[6] Liu Y., Yuan H. C., Lin D. D. (2000). Studies on risk factors for liver fibrosis of Schistosomiasis japonica. *Zhongguo ji sheng chong xue yu ji sheng chong bing za zhi = Chinese journal of parasitology & parasitic diseases*.

[7] Feng Y., Liang Y., Ren J., Dai C. (2018). Canonical Wnt signaling promotes macrophage proliferation during kidney fibrosis. *Kidney diseases*.

[8] Hou J., Shi J., Chen L. (2018). M2 macrophages promote myofibroblast differentiation of LR-MSCs and are associated with pulmonary fibrogenesis. *Cell Communication and Signaling*.

[9] Smigiel K. S., Parks W. C. (2018). Macrophages, wound healing, and fibrosis: recent insights. *Current Rheumatology Reports*.

[10] Tacke F., Zimmermann H. W. (2014). Macrophage heterogeneity in liver injury and fibrosis. *Journal of Hepatology*.

[11] Weng S.-Y., Wang X., Vijayan S. (2018). IL-4 receptor alpha signaling through macrophages differentially regulates liver fibrosis progression and reversal. *eBioMedicine*.

[12] Wermuth P. J., Jimenez S. A. (2015). The significance of macrophage polarization subtypes for animal models of tissue fibrosis and human fibrotic diseases. *Clinical and Translational Medicine*.

[13] Martinez F. O., Helming L., Gordon S. (2009). *Annual review of immunology*.

[14] Misharin A. V., Morales-Nebreda L., Reyfman P. A. (2017). Monocyte-derived alveolar macrophages drive lung fibrosis and persist in the lung over the life span. *Journal of Experimental Medicine*.

[15] Ran L., Yu Q., Zhang S. (2015). Cx3cr1 deficiency in mice attenuates hepatic granuloma formation during acute schistosomiasis by enhancing the M2-type polarization of macrophages. *Disease Models & Mechanisms*.

[16] Thompson R. W., Pesce J. T., Ramalingam T. (2008). Cationic amino acid transporter-2 regulates immunity by modulating arginase activity. *PLoS Pathogens*.

[17] Yao Y., Wang Y., Zhang Z. (2016). Chop deficiency protects mice against bleomycin-induced pulmonary fibrosis by attenuating M2 macrophage production. *Molecular Therapy*.

[18] Liu S. H., Wu C. T., Huang K. H. (2016). C/EBP homologous protein (CHOP) deficiency ameliorates renal fibrosis in unilateral ureteral obstructive kidney disease. *Oncotarget*.

[19] DeZwaan-McCabe D., Riordan J. D., Arensdorf A. M., Icardi M. S., Dupuy A. J., Rutkowski D. T. (2013). The stress-regulated transcription factor CHOP promotes hepatic inflammatory gene expression, fibrosis, and oncogenesis. *Plos Genetics*.

[20] Luo J., Liang Y., Kong F. (2017). Vascular endothelial growth factor promotes the activation of hepatic stellate cells in chronic schistosomiasis. *Immunology and cell biology*.

[21] Lenna S., Trojanowska M. (2012). The role of endoplasmic reticulum stress and the unfolded protein response in fibrosis. *Current Opinion in Rheumatology*.

[22] Paridaens A., Raevens S., Devisscher L. (2017). Modulation of the unfolded protein response by tauroursodeoxycholic acid counteracts apoptotic cell death and fibrosis in a mouse model for secondary biliary liver fibrosis. *International Journal of Molecular Sciences*.

[23] Tanjore H., Lawson W. E., Blackwell T. S. (2013). Endoplasmic reticulum stress as a pro-fibrotic stimulus. *Biochimica et Biophysica Acta-Molecular Basis of Disease*.

[24] Yang Y., Liu L., Naik I., Braunstein Z., Zhong J., Ren B. (2017). Transcription factor C/EBP homologous protein in health and diseases. *Frontiers in Immunology*.

[25] Yu Y. R., Ni X. Q., Huang J., Zhu Y. H., Qi Y. F. (2016). Taurine drinking ameliorates hepatic granuloma and fibrosis in mice infected with Schistosoma japonicum. *International Journal for Parasitology: Drugs and Drug Resistance*.

[26] Zhang L., Wang Y., Pandupuspitasari N. S. (2017). Endoplasmic reticulum stress, a new wrestler, in the pathogenesis of idiopathic pulmonary fibrosis. *American Journal of Translational Research*.

[27] Burman A., Lawson W. E., Blackwell T. S., Tanjore H. (2016). Hypoxia worsens pulmonary fibrosis through expression Of C/ebp homologous protein (chop). *American Journal of Respiratory and Critical Care Medicine*.

[28] Toriguchi K., Hatano E., Tanabe K. (2014). Attenuation of steatohepatitis, fibrosis, and carcinogenesis in mice fed a methionine-choline deficient diet by CCAAT/enhancer-binding protein homologous protein deficiency. *Journal of gastroenterology and hepatology*.

[29] Taki K., Ohmuraya M., Hashimoto D. (2015). Abstract 897: CHOP-deficiency promotes chronic inflammation-induced pancreatic fibrosis. *Cancer Research*.

[30] Tamaki N., Hatano E., Taura K. (2008). CHOP deficiency attenuates cholestasis-induced liver fibrosis by reduction of hepatocyte injury. *American Journal of Physiology. Gastrointestinal and Liver Physiology*.

[31] Vij N., Amoako M. O., Mazur S., Zeitlin P. L. (2008). CHOP transcription factor mediates IL-8 signaling in cystic fibrosis bronchial epithelial cells. *American Journal of Respiratory Cell and Molecular Biology*.

[32] San-Miguel B., Crespo I., Sanchez D. I. (2015). Melatonin inhibits autophagy and endoplasmic reticulum stress in mice with carbon tetrachloride-induced fibrosis. *Journal of Pineal Research*.

[33] Mueller K., Sunami Y., Stuetzle M. (2013). CHOP-mediated hepcidin suppression modulates hepatic iron load. *Journal of Pathology*.

[34] Liu R., Li X., Huang Z. (2018). C/EBP homologous protein-induced loss of intestinal epithelial stemness contributes to bile duct ligation-induced cholestatic liver injury in mice. *Hepatology*.

[35] Kim Y., Natarajan S. K., Chung S. (2018). Gamma-tocotrienol attenuates the hepatic inflammation and fibrosis by suppressing endoplasmic reticulum stress in mice. *Molecular Nutrition & Food Research*.

[36] Tan T. C. H., Crawford D. H. G., Jaskowski L. A. (2013). Excess iron modulates endoplasmic reticulum stress-associated pathways in a mouse model of alcohol and high-fat diet-induced liver injury. *Laboratory Investigation*.

[37] Xu J., Lai K. K. Y., Verlinsky A. (2011). Synergistic steatohepatitis by moderate obesity and alcohol in mice despite increased adiponectin and p-AMPK. *Journal of Hepatology*.

[38] Ji C., Mehrian-Shai R., Chan C., Hsu Y.-H., Kaplowitz N. (2005). Role of CHOP in hepatic apoptosis in the murine model of intragastric ethanol feeding. *Alcoholism, clinical and experimental research*.

[39] Yuan D., Xiang T., Huo Y. (2018). Preventive effects of total saponins of Panax japonicus on fatty liver fibrosis in mice. *Archives of Medical Science*.

[40] Tanaka Y., Ishitsuka Y., Hayasaka M. (2015). The exacerbating roles of CCAAT/enhancer-binding protein homologous protein (CHOP) in the development of bleomycin-induced pulmonary fibrosis and the preventive effects of tauroursodeoxycholic acid (TUDCA) against pulmonary fibrosis in mice. *Pharmacological research*.

[41] Wu J., Zhang R., Torreggiani M. (2010). Induction of Diabetes in Aged C57B6 Mice Results in Severe Nephropathy: An Association with Oxidative Stress, Endoplasmic Reticulum Stress, and Inflammation. *The American journal of pathology*.

[42] Schramm G., Gronow A., Knobloch J. (2006). IPSE/alpha-1: A major immunogenic component secreted from Schistosoma mansoni eggs. *Molecular and biochemical parasitology*.

[43] Everts B., Perona-Wright G., Smits H. H. (2009). Omega-1, a glycoprotein secreted by Schistosoma mansoni eggs, drives Th2 responses. *The Journal of experimental medicine*.

[44] Schramm G., Hamilton J. V., Balog C. I. A. (2009). Molecular characterisation of kappa-5, a major antigenic glycoprotein from Schistosoma mansoni eggs. *Molecular and biochemical parasitology*.

[45] Sotillo J., Pearson M. S., Becker L. (2019). In-depth proteomic characterization of Schistosoma haematobium: towards the development of new tools for elimination. *PLoS Neglected Tropical Diseases*.

[46] De Marco Verissimo C., Potriquet J., You H., McManus D. P., Mulvenna J., Jones M. K. (2019). Qualitative and quantitative proteomic analyses of Schistosoma japonicum eggs and egg-derived secretory-excretory proteins. *Parasites & Vectors*.

[47] Gong W., Huang F., Sun L. (2018). Toll-like receptor-2 regulates macrophage polarization induced by excretory-secretory antigens from Schistosoma japonicum eggs and promotes liver pathology in murine schistosomiasis. *PLoS neglected tropical diseases*.

[48] Du P., Ma Q., Zhu Z.-D. (2016). Mechanism of Corilagin interference with IL-13/STAT6 signaling pathways in hepatic alternative activation macrophages in schistosomiasis-induced liver fibrosis in mouse model. *European journal of pharmacology*.

[49] He X., Tang R., Sun Y. (2016). MicroR-146 blocks the activation of M1 macrophage by targeting signal transducer and activator of transcription 1 in hepatic schistosomiasis. *EBioMedicine*.

[50] Duffield J. S., Lupher M., Thannickal V. J., Wynn T. A. (2013). Host responses in tissue repair and fibrosis. *Annual Review of Pathology: Mechanisms of Disease*.

[51] Hams E., Aviello G., Fallon P. G. (2013). The schistosoma granuloma: friend or foe?. *Frontiers in immunology*.

[52] Joshi S., Singh A. R., Wong S. S. (2017). Rac2 is required for alternative macrophage activation and bleomycin induced pulmonary fibrosis; a macrophage autonomous phenotype. *PloS one*.

[53] Shen B., Liu X., Fan Y., Qiu J. (2014). Macrophages regulate renal fibrosis through modulating TGF*β* superfamily signaling. *Inflammation*.

[54] Pan B., Liu G., Jiang Z., Zheng D. (2015). Regulation of renal fibrosis by macrophage polarization. *Cellular Physiology and Biochemistry*.

[55] Sung S. A., Jo S. K., Cho W. Y., Won N. H., Kim H. K. (2007). Reduction of renal fibrosis as a result of liposome encapsulated clodronate induced macrophage depletion after unilateral ureteral obstruction in rats. *Nephron. Experimental Nephrology*.

[56] Ayaub E. A., Kolb P. S., Mohammed-Ali Z. (2016). GRP78 and CHOP modulate macrophage apoptosis and the development of bleomycin-induced pulmonary fibrosis. *Journal of Pathology*.

[57] Zhang M., Guo Y., Fu H. (2015). Chop deficiency prevents UUO-induced renal fibrosis by attenuating fibrotic signals originated from Hmgb1/TLR4/NF *κ* B/IL-1 *β* signaling. *Cell Death & Disease*.

[58] Rahal O. M., Wolfe A. R., Mandal P. K. (2018). Blocking interleukin (IL)4- and IL13-mediated phosphorylation of STAT6 (Tyr641) decreases M2 polarization of macrophages and protects against macrophage-mediated radioresistance of inflammatory breast cancer. *International Journal of Radiation Oncology, Biology, Physics*.

[59] Gong M., Zhuo X., Ma A. (2017). STAT6 upregulation promotes M2 macrophage polarization to suppress atherosclerosis. *Medical Science Monitor Basic Research*.

[60] Moreno J. L., Mikhailenko I., Tondravi M. M., Keegan A. D. (2007). IL-4 promotes the formation of multinucleated giant cells from macrophage precursors by a STAT6-dependent, homotypic mechanism: contribution of E-cadherin. *Journal of Leukocyte Biology*.

[61] Sanson M., Distel E., Fisher E. A. (2013). HDL induces the expression of the M2 macrophage markers arginase 1 and Fizz-1 in a STAT6-dependent process. *PloS one*.

[62] Takeda K., Kamanaka M., Tanaka T., Kishimoto T., Akira S. (1996). Impaired IL-13-mediated functions of macrophages in STAT6-deficient mice. *Journal of immunology*.

[63] Adedokun S. A., Seamans B. N., Cox N. T. (2018). Interleukin-4 and STAT6 promoter polymorphisms but not interleukin-10 or 13 are essential for schistosomiasis and associated disease burden among Nigerian children. *Infection Genetics and Evolution*.

[64] Yan J., Zhang Z., Yang J., Mitch W. E., Wang Y. (2015). JAK3/STAT6 stimulates bone marrow-derived fibroblast activation in renal fibrosis. *Journal of the American Society of Nephrology : JASN*.

[65] Nikota J., Banville A., Goodwin L. R. (2017). Stat-6 signaling pathway and not Interleukin-1 mediates multi-walled carbon nanotube-induced lung fibrosis in mice: insights from an adverse outcome pathway framework. *Particle and fibre toxicology*.

[66] Shirey K. A., Pletneva L. M., Puche A. C. (2010). Control of RSV-induced lung injury by alternatively activated macrophages is IL-4R*α*-, TLR4-, and IFN-*β*-dependent. *Mucosal Immunology*.

[67] Liang H., Zhang Z., Yan J. (2017). The IL-4 receptor *α* has a critical role in bone marrow-derived fibroblast activation and renal fibrosis. *Kidney international*.

[68] Knipper J. A., Willenborg S., Brinckmann J. (2015). Interleukin-4 receptor *α* signaling in myeloid cells controls collagen fibril assembly in skin repair. *Immunity*.

[69] Wang Y., Zhu J., Zhang L. (2017). Role of C/EBP homologous protein and endoplasmic reticulum stress in asthma exacerbation by regulating the IL-4/signal transducer and activator of transcription 6/transcription factor EC/IL-4 receptor *α* positive feedback loop in M2 macrophages. *Journal of Allergy and Clinical Immunology*.

[70] Liao X., Sharma N., Kapadia F. (2011). Krüppel-like factor 4 regulates macrophage polarization. *The Journal of clinical investigation*.

[71] Xue Y.-k., Tan J., Dou D.-w. (2016). Effect of Kruppel-like factor 4 on Notch pathway in hepatic stellate cells. *Journal of Huazhong University of Science and Technology [Medical Sciences]*.

[72] Men R., Wen M., Zhao M. (2017). MircoRNA-145 promotes activation of hepatic stellate cells via targeting Kruppel-like factor 4. *Scientific Reports*.

[73] Satoh T., Takeuchi O., Vandenbon A. (2010). The Jmjd3-Irf4 axis regulates M2 macrophage polarization and host responses against helminth infection. *Nature immunology*.

[74] Ge S., Zhang L., Xie J. (2016). MicroRNA-146b regulates hepatic stellate cell activation via targeting of KLF4. *Annals of hepatology*.

[75] Tao L., Ma W., Wu L. (2019). Glial cell line-derived neurotrophic factor (GDNF) mediates hepatic stellate cell activation via ALK5/Smad signalling. *Gut*.

[76] Han J., Zhang X., Lau J. K. (2019). Bone marrow-derived macrophage contributes to fibrosing steatohepatitis through activating hepatic stellate cells. *The Journal of Pathology*.

[77] Zhou A.-X., Wang X., Lin C. S. (2015). C/EBP-homologous protein (CHOP) in vascular smooth muscle cells regulates their proliferation in aortic explants and atherosclerotic lesions. *Circulation research*.

[78] Handa P., Thomas S., Morgan-Stevenson V. (2019). Iron alters macrophage polarization status and leads to steatohepatitis and fibrogenesis. *Journal of Leukocyte Biology*.

