# Supplementary material for: C/EBP Homologous Protein (CHOP) Activates Macrophages and Promotes Liver Fibrosis in Schistosoma japonicum-Infected Mice
Source: J Immunol Res. 2019 Dec 1;2019:5148575. doi: 10.1155/2019/5148575 (PMC6914929; doi:10.1155/2019/5148575)
Supplement: Supplementary Materials — Supplementary Figure 1: H&E staining and Masson staining of liver tissue of mice. (A) Liver tissues were stained with HE in uninfected group mice. (B) Liver tissues were stained with HE after 10 weeks of S. japonicum infection. (C) Liver tissues were stained with Masson's trichrome staining after 10 weeks of S. japonicum infection. The thin black arrow indicates fibres and the thick black arrow indicates schistosome eggs (magnified 50 times, 100 times, 200 times, and 400 times from left to right). Supplementary Figure 2: negative control for immunofluorescence. (A) Negative control of liver immunofluorescence in uninfected group mice. (B) Negative control of liver immunofluorescence after 10 weeks of S. japonicum infection (magnified by 200 times (left) and 400 times (right)). Supplementary Figure 3: negative control for immunohistochemistry. (A) Negative control of liver immunohistochemistry in uninfected group mice. (B) Negative control of liver immunohistochemistry after 10 weeks of S. japonicum infection (magnified by 200 times (left) and 400 times (right)). [file 5148575.f1.docx]

Supplementary materials description of manuscript 5148575


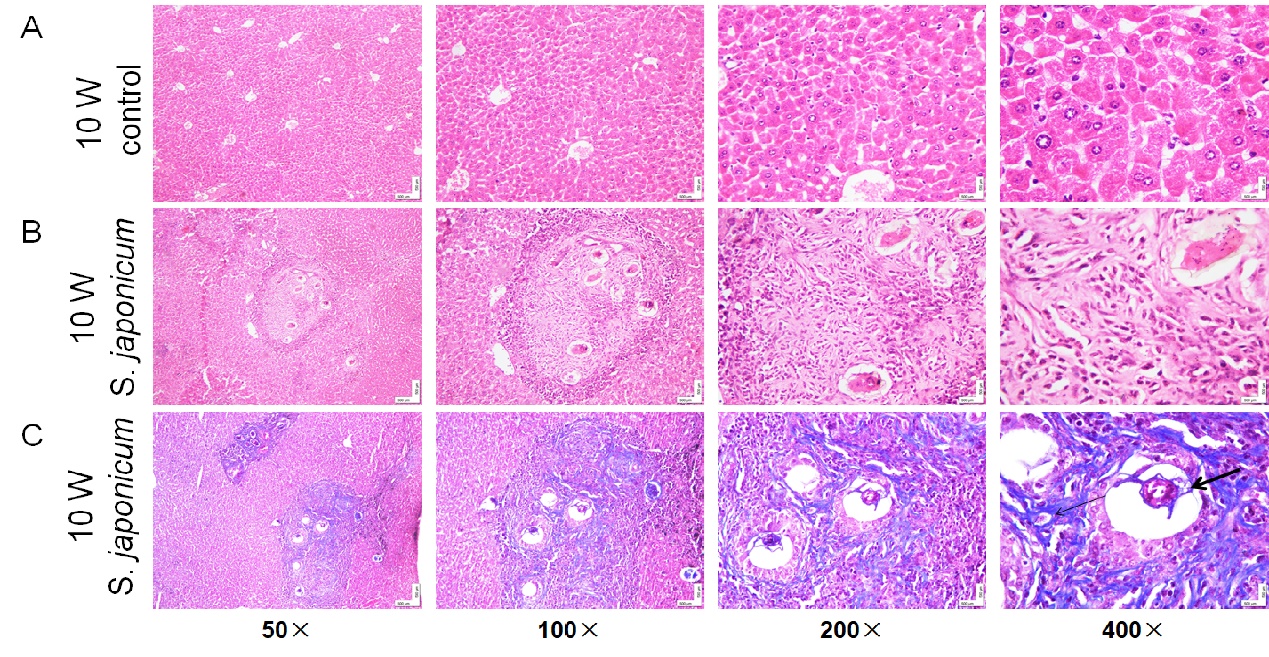


Supplementary figure 1. H&E staining and Masson staining of liver tissue of mice. (A) Liver tissues were stained with HE in uninfected group mice. (B) Liver tissues were stained with HE after 10 weeks of *S. japonicum* infection. (C) Liver tissues were stained with Masson’s trichrome staining after 10 weeks of *S. japonicum* infection. Thin black arrow indicates fibers and thick black arrow indicates schistosome eggs. (Magnified 50 times, 100 times, 200 times, and 400 times from left to right.).


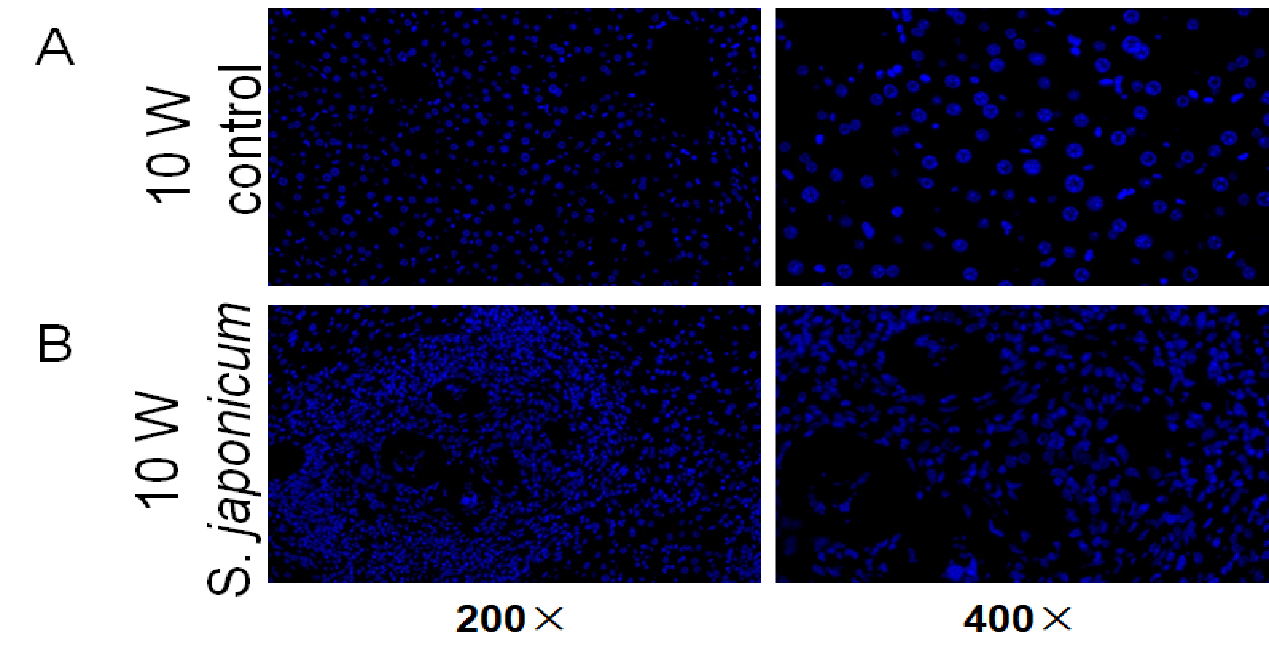


Supplementary figure 2. Negative control for immunofluorescence. (A) Negative control of liver immunofluorescence in uninfected group mice. (B) Negative control of liver immunofluorescence after 10 weeks of *S. japonicum* infection. (magnified by 200 times (left) and 400 times (right), respectively.)


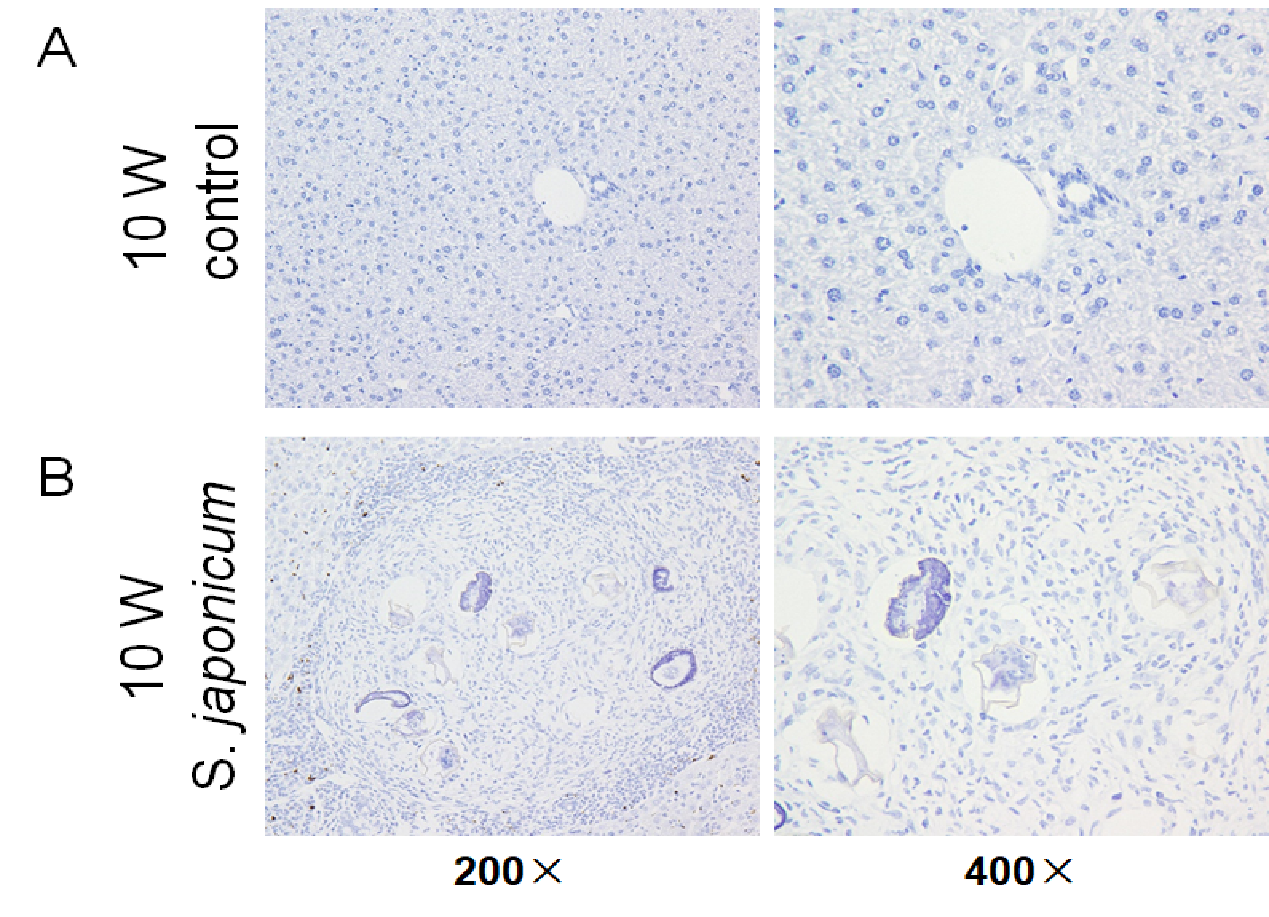


Supplementary figure 3. Negative control for immunohistochemistry. (A) Negative control of liver immunohistochemistry in uninfected group mice. (B) Negative control of liver immunohistochemistry after 10 weeks of S. japonicum infection. (magnified by 200 times (left) and 400 times (right), respectively.)
